# Factors influencing the injury severity score and the probability of survival in patients who fell from height

**DOI:** 10.1038/s41598-021-95226-w

**Published:** 2021-07-30

**Authors:** Masashi Fujii, Tsutomu Shirakawa, Mami Nakamura, Mineko Baba, Masahito Hitosugi

**Affiliations:** 1 Department of Anaesthesia, Nagahama Red Cross Hospital, 14-7 Miyamae, Nagahama, Otsu, Shiga 526-8585 Japan; 2 Department of Orthopaedic Surgery, Nagahama Red Cross Hospital, Nagahama, Shiga Japan; 3 Department of Legal Medicine, Shiga University of Medical Science, Otsu, Shiga Japan; 4 Centre for Integrated Medical Research, Keio University School of Medicine, Tokyo, Japan

**Keywords:** Medical research, Risk factors

## Abstract

In Japan, falls from height result in the second highest trauma mortality rate after traffic motor vehicle collisions and the highest trauma-related mortality rate amongst young people. We aimed to identify factors that worsen injury severity and lower survival probability of patients who fell from height and to contribute to the improvement of their prehospital and in-hospital care. This retrospective analysis retrieved hospital records of 179 patients aged ≥ 15 years who were transported to our hospital after a fall from height during April 2014–March 2020. On multiple regression analysis, fall height ≥ 5 m more significantly resulted in higher the injury severity score. Logistic regression analysis revealed that fall height ≥ 5 m with the reference of 2–3 m significantly resulted in lower the survival probability with odds ratio (95% confidence interval) of 0.10 (0.02–0.55). Using ‘feet-first’ as the reference body position, the odds ratios (95% confidence interval) of survival for those who impacted the surface on the lateral or dorsal regions were 0.11 (0.02–0.64) and 0.17 (0.03–0.99), respectively. Collecting information on the abovementioned factors at pre-hospitalisation may facilitate prompt diagnosis and treatment. These results may help improve prehospital and in-hospital care, avoiding preventable trauma deaths.

## Introduction

Falls from height result in the second highest trauma-related mortality rate after motor vehicle collisions and are the most common cause of trauma-related mortality amongst young people in Japan^1^. In addition to establishing preventive measures, improving the prehospital and in-hospital care of patients who fell from heights may be necessary to decrease their mortality rate. Various factors are related to the aggravation of the condition of patients who fell from height; amongst these factors, injury type and severity should be carefully considered when treating these patients. Some studies have investigated factors that affect the mortality of patients who fell from height and concluded that fall height, impact surface properties, alcohol consumption, psychiatric medicine use, suicide attempt, and body position at the time of surface impact worsen mortality rates^2–5^. However, because of the small number of studies, these results have not been fully confirmed.

Most patients who fell from height do not present to medical facilities at a critical stage with cardiopulmonary arrest; thus, avoiding preventable trauma deaths represents the highest priority. If the factors that result in greater anatomical injury severity can be identified and evaluated pre-hospitalisation, they may facilitate triage and contribute to improving the mortality rate by allowing the appropriate selection of hospitals for individual patients. In addition, if the factors that influence the probability of survival (Ps) are clarified, they may help improve the quality of trauma treatment, particularly the initial response. To the best of our knowledge, no study has examined the factors that affect injury severity or Ps of patients who fell from height. Therefore, this study aimed to identify factors that resulting in higher injury severity and lower Ps of patients who fell from height and to contribute to the improvement of prehospital and in-hospital care. Furthermore, to find the characteristics of suicidal cases, characteristics of those that fell from height were compared between individuals with suicide attempt and without.

## Methods

### Subjects

Nagahama Red Cross Hospital is located in a city with a population of approximately 120,000 and is the only emergency medical centre in northern Shiga Prefecture in Japan. Our hospital accepts approximately 23,000 emergency patients, including > 3700 ambulance-transported patients annually. Between April 2014 and March 2020, 280 patients were transported to our hospital after a fall from a height of 2 m or greater^6^. Only falls in which the body was completely suspended in the air were considered based on their medical records. Amongst these patients, 58 who did not free fall (e.g. fell down stairs), 17 who fell whilst riding on a vehicle, 2 who fell on snow, and 24 aged less than 14 years were excluded. The remaining 179 patients were included in the analyses.

This study was conducted with the approval of the Institutional Review Board of Nagahama Red Cross Hospital (approval number: 2020-030) and with due consideration given to the protection of personal information based on the Declaration of Helsinki of Human Rights.

Informed consent was obtained from all subjects or, if subjects are under 20, from a patient and/or legal guardian.

### Assessments

The hospital records of all enrolled patients were reviewed and, besides general information (e.g. age and sex), the following parameters were examined:

- Injury Severity Score (ISS): The ISS is an index of the anatomical severity of trauma^7^. The body was divided into the head, face, neck, chest, abdomen, spine, limbs, and body surface, and the severity of trauma at each site was evaluated on a scale of 0‒6 points^8^. We subsequently summed the squares of the scores of each of the three highest-scoring items to determine the ISS. The ISS total score ranges from 0 to 75, and a score of 15 or higher is considered to indicate serious or severe trauma, signalling the need for careful treatment.
- Ps: The Ps was calculated based on vital signs or the ISS at the time of patient arrival^9^. If the patient died with a Ps > 0.5, death was considered avoidable. If the patient died with a 0.25 ≤ Ps ≤ 0.5, the death could have been avoided (preventable trauma death). If the patient died with a Ps < 0.25, it was considered a non-preventable death. We classified Ps as ≥ 0.9 or < 0.9 for analysis.
- Date: We classified the date of the fall as a weekday or a weekend or holiday.
- Time: We classified the time of the fall as daytime or after sunset.
- Fall height: We classified fall heights as 2 to < 3 m, 3 to < 5 m, and ≥ 5 m. If the person’s testimony could not be obtained, we interviewed and comprehensively investigated the ambulance crew and police to determine this information.
- Suicide attempt: We investigated whether the cause of the fall was a suicide attempt.
- Alcohol consumption: We determined whether the patient had been drinking alcohol at the time of the fall based on blood alcohol levels.
- Psychiatric medication: We used medical records to investigate whether the patient was taking psychiatric medication.
- Anticoagulant use: We used medical records to investigate whether the patient was taking anticoagulant.
- Properties of the impact surface: We investigated the ground at the fall site and classified it into three categories: indoor, soft ground (soil and lawn), and hard ground (asphalt, concrete).
- Surgery: We investigated whether the patient had any surgery during hospitalisation after the fall.
- Helmet use: We noted whether the patient was wearing a helmet at the time of the fall.
- Body position at the time of surface impact: We established the parts of the body that were most impacted during the fall. That is, the injury mechanism was determined based on the information at the time of the fall and findings of damage obtained by physical and image examinations. The most impacted regions were classified into five categories (from minor to major injury): lower limbs, dorsal region, ventral region, lateral region, and head.

For transfer patients, we investigated vital signs and the fall context as much as possible. Unknown data were considered missing values.

### Statistical analyses

The data for categorical variables are summarised as a ratio or frequency. In the case of continuous variables, the mean and standard deviation values were used for normally distributed data, and the median and interquartile range values were used for non-normally distributed data. The chi-squared test was used to compare frequencies between two groups. To determine the significance of the differences between two groups, we used the *t*-test for normally distributed data and the Mann‒Whitney *U* test for non-normally distributed data.

First, factors that affected the ISS were examined based on the situation at the time of occurrence and history of the patient. Univariate regression analysis was performed based on sex, age, weight, body mass index (BMI), fall height, anticoagulants, psychiatric medication, alcohol consumption, body position at the time of surface impact, suicide attempt, properties of the impact surface, and helmet use. To identify variables that were independently associated with factors, multiple regression analysis was performed using the ISS as the outcome variable.

Second, factors with a Ps of < 90% were examined based on the conditions at the time of occurrence and the findings obtained during treatment. Univariate logistic regression analysis was performed based on sex, weight, BMI, date, time of fall, fall height, suicide attempt, alcohol consumption, psychiatric medication, properties of the impact surface, surgery, helmet use, and body position at the time of surface impact. To identify which variables were independently associated with poor Ps, a multivariate logistic regression analysis was performed.

Finally, comparisons between patients with and without suicide attempts were performed. The following characteristics were compared: sex, age, weight, BMI, date, time of fall, fall height, alcohol consumption, psychiatric medication, properties of the impact surface, surgery, helmet use, and body position at the time of surface impact.

All analyses were performed using the Statistical Package for the Social Sciences version 23 (International Business Machines Corp., Armonk, NY, USA), and a *p* value < 0.05 was considered statistically significant.

## Results

### General characteristics

Amongst the 179 patients included in this study, 151 (84.4%) were male and 28 (15.6%) were female. The median age was 57.0 (39.0‒68.0) years. The median body weight was 61.0 (55.0‒69.5) kg, and the median BMI was 22.0 (20.1‒24.5) kg/m^2^. Anticoagulants were taken by 15 patients (8.4%), and antipsychotic drugs were taken by 32 patients (17.9%). Seven patients (3.9%) were drinking alcohol at the time of the fall. There were 26 (14.5%) suicide attempts. The characteristics of the patients are shown in Table 1. Four patients died shortly after arriving at the hospital.

**Table 1:** General characteristics of all patients.

Characteristic	Total (n = 179)
Age, median (quartile), years	57.0 (39.0–68.0)
Male sex, no. (%)	151 (84.4)
Weight, median (quartile), kg	61.0 (55.0–69.5)
Body mass index, median (quartile)	22.0 (20.1–24.5)
Fall height, median, (quartile), m	3.0 (2.0–3.0)
Suicide attempt, no. (%)	26 (14.5)
Alcohol consumption, no. (%)	7 (3.9)
Psychiatric medicine use, no. (%)	32 (17.9)
Helmet use, no. (%)	14 (7.8)
Anticoagulant use (%)	15 (8.4)
Underwent surgery during hospitalisation, no. (%)	92 (51.4)
Injury severity score, median (quartile)	10 (5.0–19.0)
Probability of survival, median (quartile)	0.98 (0.95–0.99)
Date, no. (%)	
Weekday	125 (69.8)
Weekend	54 (30.2)
Time of fall, no. (%)	
Day time	154 (86.0)
After sunset	24 (13.4)
Unknown	1 (0.6)
Properties of the impact surface, no. (%)	
Indoor	14 (7.8)
Soft ground	86 (48.0)
Hard ground	68 (38.0)
Unknown	11 (6.2)
Body position at the time of surface impact, no. (%)	
Head	3 (1.7)
Ventral region	26 (14.5)
Dorsal region	56 (31.3)
Lower limbs	63 (35.2)
Lateral region	30 (16.8)
Unknown	1 (0.5)

### Factors influencing injury severity score

Univariate analysis of the factors resulting in higher the ISS revealed that fall height ≥ 5 m (*p* < 0.001), body position at the time of surface impact (impacting from the head and lateral regions, *p* value of 0.027 and 0.001, respectively), and properties of the impact surface (indoor and soft ground, *p* value of 0.039 and 0.002, respectively) were significant determinants (Table 2). Multiple regression analysis was performed using these three items as predictive variables. The factors that significantly influenced the ISS were fall height (falling from 3 to 5 m and ≥ 5 m, *p* value of 0.006 and < 0.001, respectively), body position at the time of surface impact (impacting from the head, lateral and dorsal regions, *p* value of 0.007, < 0.001 and 0.04, respectively), and soft ground in properties of the impact surface (*p* = 0.002) (Table 3).

**Table 2:** Univariate regression analysis results for the injury severity score.

Criterion	Regression coefficient	95% confidence interval		Standard regression coefficient	*p* value
Age	− 0.016	− 0.096	0.063	− 0.03	0.688
Sex	− 0.016	− 4.375	3.987	− 0.007	0.927
Weight	0.028	− 0.101	0.156	0.035	0.672
Body mass index	0.144	− 0.322	0.611	0.052	0.542
Fall height					
2 to < 3 m	Ref	Ref	Ref	Ref	Ref
3 to < 5 m	2.517	− 0.631	5.665	0.119	0.116
≥ 5 m	9.940	5.747	14.134	0.353	< 0.001
Suicide attempt	3.312	− 0.970	7.594	0.114	0.129
Alcohol consumption	2.355	− 5.631	10.341	0.049	0.561
Psychiatric medicine use	2.247	− 1.703	6.197	0.084	0.263
Helmet use	3.067	− 2.732	8.866	0.102	0.297
Anticoagulant use	− 3.557	− 9.013	1.899	− 0.096	0.20
Properties of the impact surface					
Hard ground	Ref	Ref	Ref	Ref	Ref
Indoor	− 5.987	− 11.669	− 0.306	− 0.165	0.039
Soft ground	− 4.989	− 8.130	− 1.848	− 0.248	0.002
Body position at the time of surface impact					
Lower limbs	Ref	Ref	Ref	Ref	Ref
Head	13.032	1.519	24.545	0.164	0.027
Ventral region	0.250	− 4.292	4.791	0.009	0.914
Dorsal region	1.079	− 2.499	4.658	0.049	0.552
Lateral region	7.565	3.243	11.887	0.278	0.001

**Table 3:** Multiple regression analysis results for the injury severity Score.

Criterion	Regression coefficient	95% confidence interval		Standard regression coefficient	*p* value
Fall height					
2 to < 3 m	Ref	Ref	Ref	Ref	Ref
3 to < 5 m	4.370	1.270	7.470	0.213	0.006
≥ 5 m	11.850	7.360	16.340	0.408	< 0.001
Properties of the impact surface					
Hard ground	Ref	Ref	Ref	Ref	Ref
Indoor	− 2.474	− 7.891	2.943	− 0.069	0.368
Soft ground	− 4.820	− 7.856	− 1.784	− 0.241	0.002
Body position at the time of surface impact					
Lower limbs	Ref	Ref	Ref	Ref	Ref
Head	15.046	4.212	25.881	0.200	0.007
Ventral region	2.744	− 1.564	7.052	0.098	0.210
Dorsal region	3.740	0.162	7.317	0.174	0.041
Lateral region	7.645	3.422	11.868	0.286	< 0.001

### Factors influencing the probability of survival

Univariate logistic analysis indicated that the following factors were associated with a Ps rate < 0.9: fall height (odds ratio of 0.27 for heights ≥ 5 m), properties of the impact surface (odds ratio of 2.38 for soft ground), and body position at the time of surface impact (odds ratio of 0.12 for lateral region) (Table 4). Next, multivariate logistic regression analysis was performed using these factors as predictive variables. The factors related to a significantly worse prognosis were fall height (odds ratio of 0.10 for heights ≥ 5 m) and body position at the time of surface impact (odds ratio of 0.11 and 0.17 when impacting from the lateral and dorsal regions, respectively) (Table 5).

**Table 4:** Univariate logistic regression analysis results for the probability of survival.

Criterion	Odds ratio	95% confidence interval		*p* value
Sex	3.193	0.405	25.203	0.271
Weight	0.994	0.957	1.032	0.743
Body mass index	0.940	0.819	1.079	0.379
Fall height				
2 to < 3 m	Ref	Ref	Ref	Ref
3 to < 5 m	0.526	0.184	1.503	0.230
≥ 5 m	0.265	0.073	0.954	0.042
Suicide attempt	2.080	0.258	16.783	0.492
Alcohol consumption	0.298	0.052	1.695	0.172
Psychiatric medicine use	3.193	0.405	25.203	0.271
Helmet use	0.833	0.161	4.326	0.828
Anticoagulant use	2.258	0.281	18.129	0.443
Underwent surgery during hospitalisation	0.594	0.235	1.502	0.271
Date	1.508	0.581	3.916	0.399
Time of fall	0.787	0.208	2.971	0.724
Properties of the impact surface				
Hard ground	Ref	Ref	Ref	Ref
Indoor	3.184	0.378	26.781	0.287
Soft ground	2.379	0.874	6.479	0.090
Body position at the time of surface impact				
Lower limbs	Ref	Ref	Ref	Ref
Head	0.091	0.006	1.476	0.092
Ventral region	0.333	0.052	2.143	0.247
Dorsal region	0.312	0.061	1.581	0.159
Lateral region	0.119	0.023	0.612	0.011

**Table 5:** Multivariate logistic regression analysis results for the probability of survival.

Criterion	Odds ratio	95% confidence interval		*p* value
Fall height				
2 to < 3 m	Ref	Ref	Ref	Ref
3 to < 5 m	0.312	0.095	1.028	0.056
≥ 5 m	0.1	0.018	0.554	0.008
Properties of the impact surface				
Hard ground	Ref	Ref	Ref	Ref
Indoor	1.939	0.178	21.095	0.587
Soft ground	0.555	0.055	5.628	0.618
Body position at the time of surface impact				
Lower limbs	Ref	Ref	Ref	Ref
Head	0.053	0.003	1.056	0.054
Ventral region	0.193	0.026	1.413	0.105
Dorsal region	0.169	0.029	0.992	0.049
Lateral region	0.109	0.019	0.640	0.014

### Comparison between patients with and without suicide attempts

Compared with patients who did not attempt suicide, those who attempted suicide were significantly younger (*p* < 0.001), had a higher proportion of female patients (*p* < 0.001), weighed less (*p* = 0.031), had a lower BMI (*p* = 0.025), used more oral antipsychotic drugs (*p* < 0.001), and underwent surgery during hospitalisation more frequently (*p* < 0.001). Furthermore, a greater proportion of patients who attempted suicide fell at night-time (after sunset) (*p* < 0.001), from higher heights (*p* < 0.001), and with their lower limbs impacting the surface (*p* < 0.001) than those who did not attempt suicide (Table 6).

**Table 6:** Comparison between individuals who fell from a height in a suicide attempt and those who did not.

Characteristic	Suicide attempt (n = 26)	Non-suicide attempt (n = 153)	*p* value
Age, median (quartile), years	32.5 (19.8–39.3)	61 (46–70)	< 0.001
Male sex, no. (%)	11 (42.3)	132 (91.0)	< 0.001
Weight, median (quartile), kg	56.5 (48.0–67.8)	63 (56.0–70.0)	0.031
Body mass index, median (quartile)	20.6 (18.5–23.4)	22.1 (20.3–24.9)	0.025
Fall height, median, (quartile), metre	3.0 (3.0–9.0)	3.0 (2.0–3.0)	< 0.001
Alcohol consumption, no. (%)	2 (7.7)	5 (3.3)	0.144
Psychiatric medication use, no. (%)	23 (88.5)	9 (5.9)	< 0.001
Anticoagulants use, no. (%)	0 (0.0)	15 (9.8)	0.132
Helmet use, no. (%)	0 (0.0)	14 (9.2)	0.02
Underwent surgery during hospitalisation, no. (%)	24 (92.3)	68 (44.4)	< 0.001
Injury Severity Score, median (quartile)	16 (8.8–24.3)	10 (5.0–18.5)	0.082
Probability of survival, median (quartile)	0.98 (0.93–0.99)	0.98 (0.95–0.99)	0.104
Date, no. (%)			0.358
Weekday	16 (61.5)	109 (71.2)	
Weekend	10 (38.5)	44 (28.8)	
Time of fall, no. (%)			< 0.001
Day time	15 (57.7)	139 (90.8)	
After sunset	10 (38.5)	14 (9.2)	
Unknown	1 (3.8)	0 (0.0)	
Properties of the impact surface, no. (%)			0.701
Indoor	2 (7.7)	12 (8.3)	
Soft ground	11 (42.3)	75 (59.5)	
Hard ground	12 (46.2)	56 (39.2)	
Unknown	1 (3.8)	0 (0.0)	
Body position at the time of surface impact, no. (%)			< 0.001
Head	0 (0.0)	3 (2.0)	
Ventral region	2 (7.7)	24 (15.7)	
Dorsal region	0 (0.0)	56 (36.6)	
Lower limbs	22 (84.7)	41 (26.7)	
Lateral region	1 (3.8)	29 (19.0)	
Unknown	1 (3.8)	0 (0.0)	

## Discussion

In this study, the fall height ≥ 5 m, body position of the lateral region at the time of surface impact, and properties of the impact surface of hard ground were factors resulting in higher ISS, whilst sex, weight, BMI, suicide attempt, drinking alcohol at the time of the fall, use of psychiatric medication, and helmet use were not significant factors.

Some reports have found that fall height is not a factor aggravating the outcome of a fall from height injury^5,10^, but in our survey, as in other reports, we found that fall height was a significant factor^3,4,11,12^. The impact force is defined by mass (kg) × acceleration (m/s^2^). In other words, it is defined by body weight (kg) × (speed immediately before collision − speed at impact/time to impact [s]). Thus, body weight (kg) × (√2 × √gravitational acceleration × √height of fall − 0) ÷ time to impact (s). These equations indicate that the fall height greatly contributes to the energy received by the body at impact after falling. In our study, body weight and BMI did not significantly influence ISS; thus, we hypothesised that the difference in fall height is more related to the change in impact force in the calculation formula than to the difference in body weight or BMI. In addition, the hardness of the impact surface is related to the ‘time to impact’, but since this time is extremely short, even a small difference in this time contributes greatly to the change in impact force, as expected from the above equation. Our results reflected these findings.

Body position at the time of surface impact was also a significant factor affecting both ISS and Ps. Few studies have assessed whether body position at the time of surface impact affects outcome severity^2^. Generally, severely injured patients lose consciousness and memory of events and are thus less likely to provide reliable testimony. Furthermore, the fall is often not witnessed in real time, which makes it difficult to verify how the fall occurred. Therefore, obtaining data regarding the body position at the time of impact is challenging. Our investigation undertook a novel approach in which we aimed to determine the body position at the time of impact by examining patients’ injuries and obtaining patients and witnesses’ testimonies. As expected, we observed that impacting the surface feet first resulted in the best prognosis. Furthermore, we determined that impacting the surface with the lateral or dorsal regions of the body resulted in poor prognoses. To the best of our knowledge, our study is the first to report this finding. Our finding that the body position at the time of surface impact was an important factor means that the information that can be collected even during the pre-hospitalisation period can predict the severity of a patient’s injuries. This can greatly contribute to transportation to appropriate medical institutions and implementation of proper prehospital care.

In our study, factors that resulted in lower Ps were the fall height more than 5 m, body position of the lateral region at the time of surface impact. Given that our hospital is located in a provincial city and there are few high-rise buildings around it, the height of the fall is on an average lower than those in previous reports^13–15^. However, the fact that the fall height was found to be a factor resulting in lower Ps suggests that a slight difference in height would contribute to aggravation of outcomes, even for falls from a relatively low height. Moreover, because the body position at the time of surface impact was found to be a factor influencing the prognosis, this means that even if the height is low, the prognosis becomes more severe depending on the falling position, which should therefore be recognised as important information.

Many studies have reported that the fall height in a suicide attempt is one of the main factors contributing to lower mortality rates^13,14,16^. Accordingly, we observed that patients who attempted suicide fell from greater heights than those who did not. Furthermore, patients who attempted suicide fell at night-time (after sunset) and underwent surgery more frequently than those who did not. Therefore, initially, the prognosis and ISS values seemed much worse for those with suicide attempts than for their counterparts. However, mitigating factors such as younger age, lower body weight, and a higher rate of impact on the lower limbs outweighed these aggravating factors and led to better ISS values and prognoses in patients who attempted suicide compared with those we expected^5,17,18^. Therefore, a suicide attempt was not deemed an aggravating or a prognostic factor with respect to the ISS. Accordingly, the ISS values of those with suicide attempts and those without showed no significant differences.

Furthermore, other studies have reported falls from greater heights in suicide attempt cases than those observed in our study^13,14^. This difference may be attributed to location characteristics and differences in access to high-rise buildings. After a certain fall height threshold, mitigating factors that may improve prognosis have less of an effect on the outcome, suggesting that factors other than height had a greater influence on the outcomes of individuals who fell from lower heights, i.e. the shorter the fall height, the more important are the mitigating factors. Furthermore, considering that individuals with suicide attempts have a high probability of lower-limb impact and falls from a median height of 3 m, the suicide attempt may actually represent a ‘cry for help’ instead of a resolute attempt to die. In the future, further studies should consider this possibility for patients with mental illnesses when evaluating the management of the underlying disease.

This study had several limitations. First, the mortality rate was low, and it was impossible to determine the causative factor using the mortality rate as an objective factor. Therefore, in our study, instead of the mortality rate, we used the Ps as an objective factor to investigate factors aggravating outcomes. Since the Ps was high overall, we decided to explore factors that led to a Ps < 0.9. It was considered remarkable that we could identify factors that became more severe in the surviving group. However, in the future, it will be necessary to examine factors that lead to an even lower Ps. Second, because prognosis was only predicted using the Ps in this study, the actual long-term prognosis could not be investigated; in other words, it was not possible to clarify factors that affected quality of life, such as aftereffects. Further evaluation is needed to elucidate them. Third, we did not measure blood concentrations of drugs and only assessed the use of oral medication. Given that the use of drugs may cause a fall, it will be necessary to determine the serum concentrations of neuropsychiatric drugs to establish the association between drug use and falls in the future.

In conclusion, the predominant factors that exacerbated the anatomical severity of high-altitude fall trauma in this study were the fall height ≥ 5 m, body position of the lateral region at the time of surface impact, and properties of the impact surface of hard ground, whilst the fall height more than 5 m and body position of the lateral region at the time of surface impact affected the Ps. Collecting the above information pre-hospitalisation may facilitate prompt diagnosis and treatment. These results might be useful for promoting preventive measures toward decreasing the number of fatalities caused by falls from a height.

## Data Availability

The data presented in this study are available on request from the corresponding author.
